# Cancer Pain and Non-Invasive Brain Stimulation—A Narrative Review

**DOI:** 10.3390/medicina59111957

**Published:** 2023-11-06

**Authors:** Valentina-Fineta Chiriac, Daniel Ciurescu, Daniela-Viorica Moșoiu

**Affiliations:** 1 Departament of Medical Oncology, “Dr Pompei Samarian” County Emergency Hospital, 910071 Călărași, Romania; 2 Faculty of Medicine, Transilvania University, 500036 Brașov, Romania; 3 HOSPICE Casa Sperantei, 500074 Brașov, Romania

**Keywords:** pain, neoplasm, non-invasive brain stimulation, repetitive transcranial magnetic stimulation (rTMS), transcranial direct current stimulation (tDCS), cranial electric stimulation (CES)

## Abstract

*Background and Objectives*: Pain is the most prevalent symptom in cancer patients. There is a paucity of data regarding non-invasive brain stimulation (NIBS) for the treatment of chronic pain in patients with cancer. The purpose of this article is to review the techniques of NIBS and present the published experiences of the oncological population. *Materials and Methods*: Databases including MEDLINE, Scopus, Web of Science, and the Cochrane Library were searched for articles on cancer patients with pain that was managed with non-invasive brain stimulation techniques. We included articles in English that were published from inception to January 2023. As studies were limited in number and had different designs and methodologies, a narrative review was considered as the best option to integrate data. *Results*: Four studies focusing on transcranial magnetic stimulation, six articles on transcranial direct current stimulation, and three articles regarding cranial electric stimulation were found and reviewed. *Conclusions*: Data are limited and not robust. Further studies in this field are required. Guidelines on NIBS for non-malignant chronic pain conditions provide good premises for cancer-related chronic pain.

## 1. Introduction

Cancer is one of the leading causes of death, but its major social impact regards morbidity [1]. Pain affects half of patients receiving treatment and more than two-thirds of patients with metastatic or terminal cancer [1,2]. Cancer-related pain is the most frequent and feared symptom [3].

Jan Stjernsward, one of the pioneers and author of WHO analgesic ladder, had the vision in the 1980′s ‘to achieve world freedom from cancer pain by the year 2000′ [4]. Twenty-three years after that deadline, not much has changed in the management of pain.

In the light of recent advances in cancer therapy with improved disease-free survival and overall survival, pain has become even more challenging in daily practice [5,6].

Drugs are the main treatment for pain, with opioids being the most effective pharmacological treatment. The reality shows that these are insufficient in terms of efficacy. Up to 20% of patients will not obtain relief of pain, despite a constant update of pain treatment [7,8]. Moreover, pharmacological interventions have side effects that can influence patients’ awareness and self-control and all in all, can decrease quality of life [9].

The nervous system has the ability to adapt to environmental changes. This process is called neuroplasticity. Even pain is considered a “learned concept” by the International Association for the Study of Pain [10]. The implication of neural networks and neuroplasticity have been proposed as mechanisms in the search for the pathophysiology of cancer-related pain [11]. This idea provides a new possible intervention in the field of neuromodulation of cancer pain [12].

Neuromodulation relates to “the alteration of nerve activity through targeted delivery of a stimulus, such as electrical stimulation or chemical agents, to specific neurological sites in the body” [13]. Neuromodulation can be considered as a method to restore neural activity, similar to a cardiac pacemaker re-establishing cardiac rhythm. It includes spinal cord stimulation, peripheral nerve stimulation and deep brain stimulation, as well as a vast category of non-invasive brain stimulation (NIBS).

NIBS primarily includes repetitive transcranial magnetic stimulation (rTMS), transcranial electric stimulation using direct current (tDCS), and cranial electrotherapy stimulation (CES). Other new but not very well established methods are transcranial electric stimulation using alternating current (tACS), transcranial random noise stimulation (tRNS), reduced impedance non-invasive cortical electrostimulation (RINCE), and transcranial ultrasound stimulation (TUS).

The efficiency of non-invasive brain neuromodulation techniques for chronic pain has been recently investigated using an updated Cochrane database review [14]. They provide low quality evidence for single-dose high-frequency rTMS of the motor cortex as well as for tDCS. The effect on pain appears to be of short duration. However the results are not conclusive, as studies were conducted on a small number of patients with different chronic conditions [14].

Several guidelines exists and are providing rTMS and tDCS with good levels of evidence for chronic pain such as fibromyalgia, neuropathic pain, or migraine [15,16,17].

Not much is known regarding these methods in the management of cancer pain. With the exception of two articles [15,18] that each included one study, the main database reviews on NIBS in chronic pain [14,16,17] have excluded studies on cancer patients. Even though these new techniques are mentioned in articles presenting advances in cancer pain management [19,20], no comprehensive review of the existing data exists. Recently, a systematic review and meta-analysis was performed on just four trials involving non-invasive brain stimulation in patients with cancer [21].

With an understanding of the knowledge gap in this category of patients and the urgent need to improve pain management, our primary goal was to perform a systematic review on this topic. Some disadvantages were soon noted. Studies were numerically poorly represented and diverse regarding study design and methodology. We decided to remain faithful to our main idea to provide a comprehensive review of the existing data on NIBS for the management of cancer pain. Therefore, a change in study design was made. A narrative review was considered best to fit the data found.

## 2. Materials and Methods

A wide literature search was conducted at the beginning of January 2023 using the following databases: MEDLINE (via PubMed), Scopus, Web of Science (via Clarivate), Cochrane Library, LILACS, BBO (Brazilian Library of Dentistry) and other Latin American databases (via VHL Regional Portal), and multiple databases from more than 70 countries using WorldWideScience.org and the Grey Literature Database (via DANS easy).

Keywords such as ‘transcranial direct current stimulation’, ‘transcranial magnetic stimulation’, ’cranial electrical stimulation’, ‘noninvasive brain stimulation’, ‘pain’, and ‘cancer’ were used. The search strategy combined MeSH (Medical Subject Headings) terms and synonyms with significant occurrences in major databases. The search was individualized according to the specificity of each database. The Boolean operators “AND” and “OR” were used to combine the searches. The main database search strategies are presented in Supplementary Table S1.

The inclusion criteria were as follows:

1. Articles involving adult patients with histological confirmed cancer and cancer-related pain (both tumour- or treatment-related pain)
2. Articles describing studies that used non-invasive brain stimulation, including the following:
  – transcranial direct current stimulation (tDCS) or
  – transcranial magnetic stimulation (rTMS) or
  – cranial electric stimulation (CES)
  as a method of treating cancer-related pain.

The exclusion criteria were letters to the editor, reviews, and case reports.

An English language filter was applied. No time span was imposed. All articles from database inception to the time of research—January 2023—were included.

**Figure 1:**
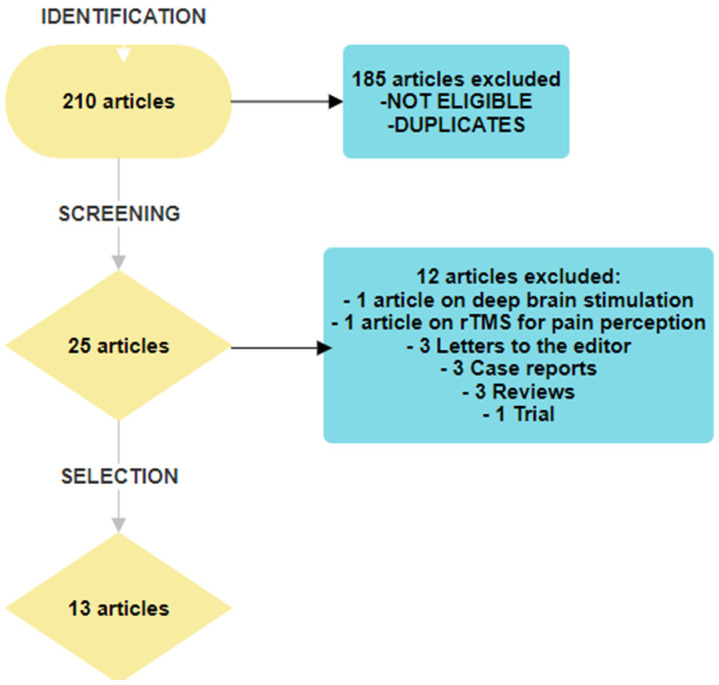
Flow chart of article selection.

The titles and abstracts found in the search were evaluated if they met the inclusion criteria. Duplicate records were automatically removed. Relevant articles were retrieved in the full text and read. A manual search was also performed on the reference lists of articles included in this study. The flow diagram is shown in Figure 1. This narrative review followed the recommendations proposed by Green on how to write narrative reviews for peer-review journals [22].

Articles that presented clinical trials were examined through the lens of the IMMPACT recommendations [23]. The domains of interest were as follows: (1) pain scoring on an 11-point scale, (2) physical functioning using the Brief Pain Inventory, (3) emotional functioning using Beck Depression Inventory or equal, (4) participant ratings of improvement and satisfaction with treatment, (5) reporting of adverse events, and (6) participant disposition (e.g., adherence to the treatment regimen and reasons for premature withdrawal from the trial). These six items were searched for in each article and if found, each was given one point. A maximum of six points was possible. The scores that each paper received can be found in Table 1, Table 2 and Table 3.

## 3. Results and Discussions

In total, 210 articles were identified. After excluding duplicates and screening for eligibility based on the title and abstract, 25 articles were selected for full-text reading. Twelve articles were excluded; one treated deep brain stimulation [34] and one discussed rTMS as a method used to study pain perception [35]. Furthermore, the following articles were excluded: three letters to the editor, three case records, three reviews, and one trial that did not consider pain as a major endpoint. The flow diagram is presented in Figure 1.

The 13 articles included were divided according to the non-invasive brain stimulation method used in cancer patients.

✧ Transcranial magnetic stimulation (rTMS)—four articles
✧ Transcranial direct current stimulation (tDCS)—six articles
✧ Cranial electric stimulation (CES)—three articles

After a short overview of each technique, articles on the topic will be discussed.

### 3.1. Transcranial Magnetic Stimulation

#### 3.1.1. Brief Technique Overview

Transcranial magnetic stimulation (TMS) was the first non-invasive brain stimulation used for research and therapeutic purposes. It uses the electric current produced by a dynamic magnetic field through electromagnetic induction. Single-pulse TMS was initially designed and used as a non-invasive method to study human pain perception [35], but its potential therapeutic role has rapidly surfaced. Repetitive TMS (rTMS) can induce long-lasting changes in brain activity. Based on this mechanism, it has been proven to be safe and well tolerated, as well as effective in numerous psychiatric and neurological conditions [17].

By enhancing neuroplasticity, rTMS has the ability to indirectly modulate central structures such as the cingulate, orbitofrontal, and prefrontal cortical regions that are implicated in pain processing [36], as well as in the pain–emotion connection [37]. It can also potentiate descending inhibitory pathways [19,38]. Another proposed mechanism is based on the effect of increasing endogenous opioids in the nervous system [39]. The implications of BDNF brain-derived neurotrophic factor (BDNF) have also been hypothesized [40].

Different sessions regarding intensity and length of time or type of impulses have been investigated from that period, and many trials have shown some benefits in chronic pain suppression [14]. The Cochrane review from 2018 indicates low and very low quality evidence for prefrontal rTMS, whereas motor cortex stimulation has been accepted as a potential method to reduce pain for short periods of time [14].

In neuropathic pain, it is considered that the damage to the peripheral nervous system implies a dysfunction in the somatosensory processing in the central nervous system [41]. rTMS has been shown to have a beneficial effect on neuropathic pain by modulating neural activity in the brain [14,42,43]. The main area of stimulation was considered the primary motor area, not the dorsolateral prefrontal cortex (DLPFC) [44]. The Cochrane systematic review and French recommendations concluded that only high-frequency stimulation (>5 Hz), with >500 stimuli and only multiple sessions, have beneficial results [14,45]. rTMS of the M1 (primary motor cortex area) contralateral to the pain side in neuropathic pain has been considered to be effective through stimulation with high frequency (>5 Hz), with level of evidence A, at a constant over the years [17,46]. A small response of rTMS to chronic pain was identified in the Cochrane review [14] with a 7% reduction in pain and quality of life. Moreover, the Latin American [15] consensus recommend level A for rTMS over M1 for fibromyalgia and neuropathic pain and level B for myofascial or musculoskeletal pain, complex regional pain, and migraine.

#### 3.1.2. Begining of rTMS in Cancer Pain

The first cases of rTMS in cancer patients were described by a French team from Nantes. In each case [37,47] good response to rTMS for refractory cancer pain was recorded. The effect was observed after a few days, with a reduction in pain, as well as a drastic reduction in analgesic drugs needed [47]. This technique has potential benefits for pain, mood, anxiety, and depression and is considered by the authors as a possible adjuvant method in the context of multidisciplinary management of palliative care [37].

#### 3.1.3. Studies Using rTMS for Cancer Pain Treatment

Four studies focusing on rTMS and cancer pain were identified (Table 1), three clinical trials [24,25,27], and one pilot study [26]. All trials received a score of four based on the IMMPACT recommendations, whereas the pilot study gathered three points.

Two studies investigated neuropathic pain [24,26], and one focused on visceral pain [25]. All four studies used the visual analogue scale (VAS) or numerical rating scale (NRS) to evaluate pain intensity. The population analyzed in this study was diverse. The first Egyptian study [24] mostly included women with neuropathic pain after mastectomy. All patients were undergoing active oncologic treatment, either chemotherapy or radiotherapy. A Japanese study [26] also investigated neuropathic pain in women, but they included patients with neuropathic pain and/or peripheral sensory neuropathy who received a chemotherapy regimen based on taxane or oxaliplatin. It is worth mentioning the different inclusion criteria for pain evaluation. The first study [24] used the DN4 score (Douleur Neuropathique 4) and included patients with a questionnaire score equal to or greater than four who were resistant to treatment for at least two months. Goto [26] defined neuropathic pain as a minimum grade 2 severity based on the National Cancer Institute Common Terminology Criteria for Adverse Events (NCI-CTCAE, version 4.0) scale. In their study, they used a combination of patient-reported symptoms and clinical assessment to select participants with chemotherapy-induced peripheral neuropathy (CIPN). One year after their previous study, the same Egyptian team published a new study [25] using the same procedure but focusing on malignant visceral pain. They included diverse oncologic localizations. The pain investigated in all cases was localized in the upper abdomen (either the right or left hypochondrium or epigastrium) and was resistant to medical treatment for at least two months or associated with significant adverse effects from medication. The latest study [27] coming from China included only lung cancer patients, with cancer pain evaluated as equal to or greater than four on the NRS. The type of pain, whether neuropathic or nociceptive, was not mentioned.

Patients with intracranial metallic devices, other metallic implanted devices (e.g., pacemakers), a history of seizures, and severe cardiac or psychiatric conditions were not permitted to participate. The number of participants was modest, with Tang [27] having the maximum (39) number of patients, whereas Khedr included 30 patients in both his studies [24,25], and Goto analyzed only 11 patients [26]. The randomized sham-controlled studies had all well-balanced groups in terms of age, duration of illness, and initial pain evaluation score. Interestingly, all patients in the two Egyptian studies [24,25] were on the same analgesic medication.

The stimulation received was also different. Khedr [24,25] used in both of his studies 10 daily sessions of rTMS at 20 Hz, applied to the motor cortical area corresponding to the hand area in the painful side or the dominant hemisphere in the case of epigastric pain. Tang [27] used a different approach, positioning the F8 coil in the dorsolateral prefrontal cortex (DLPFC), providing 1500 pulses for 15 sessions in three weeks. Goto’s pilot study [26] used four different types of stimulation, with differences in coil direction, intensity, and position, which they applied in random order to 11 patients, with one stimulation per week. A summary of the details regarding the stimulation is presented in Table 1.

The benefit was evaluated using the VAS or NRS as the principal endpoint. Moreover, the studies used the verbal descriptor scale (VDS), Hamilton scale for depression (HAD), Leeds assessment of neuropathic symptoms and signs (LANSS) [24], oral morphine equivalent (OME), quality of life [27], and McGill Pain Questionnaire 2 (SF-MPQ2) [26]. One study used blood samples to test for the level of serum human dynorphine [25].

The results of all four studies were positive. The first randomized clinical trial [24] reported the benefit of rTMS based on the VAS score but also on the decrease in medication in the treated group compared to the control group. As shown in other studies on non-cancer pain, the effect on pain appeared after several sessions and maintained some effect after the end of stimulation but was not present at the one month follow up. Interestingly, at one month, the reduction in the HAD and LANSS scores was still significant. It should be noted that participants reported no side effects. Regarding the curves depicting the effect of stimulation vs. sham, it is interesting to see that even the sham group had some benefit, with a decrease in scores. In addition, none of the scores returned to the baseline value in either group [24].

The second randomized trial [25] showed statistical significance in the primary outcome, measured using the VAS. The effect appeared after the fifth stimulation, had a maximum effect after the 10th stimulation, lasted for 15 days, and was absent at the 1-month evaluation. However, the sham group showed some improvement in the VAS and VDS scores. The HAM-D evaluation showed no significant differences between the groups over time in this study population [25].

The third study [26], published in 2020 in the *Journal of Clinical Neuroscience* by a Japanese team, was a randomized pilot trial. Their results are encouraging and demonstrate the potential of rTMS as a treatment for chemotherapy-induced neuropathy, both for pain and dysesthesia. They used different coil orientation angles and found a positive effect in both the postero-anterior (PA) 90% intensity and the lateral-medial (LM) 90% intensity orientation but not in the PA 100% or PA 90% ipsilateral. They showed an amelioration in pain or dysesthesia, mainly in the targeted extremity, and limited modification only on the D-VAS for the non-targeted extremities [26]. Being designed as a pilot trial, it left some unanswered questions. The study was complex, with pain assessment of all four extremities, but it did not provide a concise definition of dysesthesia and how patients define it. They performed a randomized trial with randomization of stimulation sessions rather than of patients. One of the drawbacks is that they did not have a control group, and the meaning for stimulation randomization was not very explicit. It must be noted that the stimulation frequency was lower than that in other studies. In addition, they provided no clear explanation as to why the two other types of coil positioning did not provide any benefit.

The most recent trial was based In China and published in 2022 [27]. Their main endpoint was a decreased pain score, which was achieved with a statistical decrease in NRS in the treatment group from the third day to the third week. Similar to previous studies [24,25], the sham group also showed a decrease in the pain score. Somewhat different from the other two trials, there was no follow-up after the end of stimulation. Secondary endpoints (oral morphine equivalent dose, quality of life, depression, and anxiety) also improved after rTMS. As predicted by Nizard [37], this technique also showed a beneficial role in mood changes, with amelioration in anxiety and depressive symptoms. This is the first study to report unpleasantness, such as scalp numbness or facial muscle twitching in two patients from the rTMS group [27].

#### 3.1.4. Conclusion Regarding rTMS in Cancer Pain

The results are encouraging, with rTMS providing benefits for pain suppression in patients with cancer. This is supported by a recent meta-analysis [21] that included two of the rTMS trials [25,27]. They showed a better effect on pain with rTMS compared to CES and tDCS, but this could be attributed to the fact that tDCS and CES have been represented with only one trial. More data are required to clearly state the role of rTMS in this category of patients. Data on the effectiveness of rTMS on neuropathic pain will hopefully come in the coming years from Colombian and Hong Kong researchers, where clinical trials are open ([NCT05480410] and [NCT04107272]).

### 3.2. Transcranial Direct Current Stimulation—tDCS

#### 3.2.1. Brief Technique Overview

Transcranial direct current stimulation is another type of non-invasive brain stimulation that uses a low-voltage electric current (maximum of 3 mA). The current is produced by a small device that operates on a battery. It is delivered to the scalp using sponge electrodes. It is safe and easy to use [48].

This technique can be used to modulate neural activity. The current is not powerful enough to produce an action potential but influences the neuronal membrane potential [49]. It has the ability to induce immediate effects with anodal stimulation depolarizing membranes and increasing cortical excitability, whereas cathodal stimulation hyperpolarizes membranes and decreases cortical excitability [49].

New studies have shown that current flows through both outer and inner cortical structures [50]. Motor cortex stimulation has the capacity to modulate the activity of other regions, such as the thalamus, ventrolateral thalamus, insula, anterior cingulate gyrus, and upper brainstem [38,51]. Other mechanisms include endogenous opioid release [38] or activation of the u-opioid system [52].

Studies have proven the efficacy of tDCS in chronic pain [16,53]. The 2018 Cochrane review included 27 studies and 747 patients with chronic pain and found a 0.82 reduction that translates to a 17% reduction in pain in the intervention group. Through a meta-analysis, they reported a positive effect of tDCS on quality of life [14]. The Latin American consensus reviewed 24 tDCS studies and provided the following recommendations: level A for anodal tDCs over M1 in fibromyalgia and level B for neuropathic pain, abdominal pain, and migraine [15]. These recommendations were once again proven in a meta-analysis in 2021 [16]. None of the mentioned guidelines and reviews included studies on chronic pain in patients with cancer.

#### 3.2.2. First Data on tDCS Efficacy in Cancer Pain

There are two published case studies [54,55] using tDCS in cancer patients as a last resort in the treatment of excruciating pain. The first case dates back to 2006 and shows the benefits of tDCS in a patient with pancreatic cancer [54]. A second complex case of bladder cancer with bone metastases had an incredible response to daily stimulation for 20 min with a 1 mA intensity for five consecutive days [55]. Based on their experience, the French team of Nguyen has opened a trial in two hospitals in Nantes, investigating the impact of tDCS on cancer pain in a randomized fashion [56].

#### 3.2.3. Studies on tDCS and Cancer Pain

A database search identified seven studies comprising two pilot studies, one proof-of-concept study, and four randomized clinical trials.

One randomized trial focused primarily on the effect of one tDCS session on cancer-induced nausea and vomiting (CINV), while pain was just one symptom assessed using the Edmonton Symptom Assessment Scale. Their results showed no differences in pain scores between the groups. The lack of benefit was explained by the need for a cumulative effect of brain stimulation on pain impact [57]. This trial is not included in the following discussion.

The first randomized placebo-controlled trial using tDCS for visceral pain was designed in Germany. Finally, it was reported as a pilot study and presented as an abstract in the 2012 World Research Congress of the European Association for Palliative Care [58]. They showed promising results; however, considering the low number of patients, no conclusions were provided.

The second pilot study was conducted by a team from Michigan, USA. Feasibility and safety of tDCS for pain management in patients undergoing chemoradiotherapy for advanced head and neck cancer was researched. A comparison of the five patients in the historical control group showed less weight loss and less dysphagia in the tDCS group. This is the first study to combine tDCS with EEG recording simultaneously [51].

The team continued their work, optimized the protocol, and published in 2022 a proof-of-concept study on two patients with head and neck cancer undergoing radio-chemotherapy [59]. In this study, they used novel devices with remote tDCS, as well as functional near-infrared spectroscopy and EEG. A strict protocol, with multiple visits, clinical measurements, questionnaires, and two neuroimaging techniques, was used to provide the maximum amount of information. With a very well-described method, this study presents a unique type of pain assessment, with both hemodynamic and neurophysiologic input, with data on connections between the bilateral prefrontal and sensory cortices. Imaging revealed a decrease in functional connections between the bilateral prefrontal cortex and sensory cortex, as well as activation of the right prefrontal cortex. With this, they provided further proof that the dorsal lateral prefrontal cortex plays a role in pain perception. Even if just a proof-of-concept study, the study of pain assessment using modern technologies provides a new and optimistic perspective on pain understanding.

Three randomized clinical trials were identified and discussed. Details, as well as the IMMPACT recommendation score, can be found in Table 2. Two of the trials [28,29] have a score of four, while Hanna’s article [30] received three points.

The types of pain analyzed in the three randomized trials were diverse, ranging from chronic visceral pain in hepatocellular carcinoma [28] and acute post thoracotomy thoracic pain [29] to neuropathic post mastectomy pain syndrome [30]. The first two trials [28,29] included mostly men, whereas the last trial included only female patients [30]. Chronic pain was defined as pain resistant to medical treatment for at least two months or associated with significant adverse effects, ref. [28], or six months of neuropathic pain in the post mastectomy region, with a DN score of >4 [30]. In the Serbian trial [29], only acute pain was considered, and patients with chronic pain conditions were excluded. The size of the study population was limited, even though the Serbian trial [29] had the largest number of patients (55). Ibrahim [28] and Hanna [30] included 40 and 30 patients, respectively. As an endpoint, the VAS was maintained as the principal measurement of pain relief. Furthermore, the studies reported results based on the VDS, HAM-D, Beck Depression Inventory, morphine dose [29], and measurements of shoulder range of motion using a digital goniometer [30].

The stimulation technique involved placing the anode in the area of the primary motor cortex of the contralateral most painful abdominal area for 30 min, over ten sessions, at 2 mA [28], or the left primary motor cortex for 20 min, over five sessions, at 1.2 mA [29], or was performed bilaterally on M1 for 20 min, over five sessions, at 2 mA [30]. The cathode is usually placed in the contralateral supraorbital region [28,29] or in the ipsilateral supraorbital region [30]. More data are presented in Table 2.

The reported results were encouraging. The Egyptian team of Kehr, in addition to TMS, also took an interest in transcranial direct current stimulation [28]. A very clear and concise results section showed an improvement in pain evaluation as a primary objective but also in the depression score as a secondary outcome for both the active treatment and the sham technique. The effect was seen in both groups starting with the fifth stimulation and lasting to the tenth and then to the one month evaluation, without ever returning to the starting base value. This was valid for both the sham and active groups, with a greater effect in the tDCS group. The difference between the sham and real groups became significant at the fifth evaluation; its maximum was seen on the 10th day of stimulation and remained significant after one month for the VDS and VAS. The effect did not last in the sham group at the last evaluation at one month. For the Hamilton rating scale for depression, a similar effect was observed, but its statistical significance was lower. They reported five mild side effects in terms of skin redness and local burning sensation.

In the Serbian trial [29] performed on patients with lung cancer who underwent thoracotomy, the effect of tDCS combined with morphine was analyzed. A complex pain management protocol for the trial should be complemented because of its rigorousness. Data analyzed from 55 patients who received more than three tDCS stimulations showed that the dose of morphine administered was lower in the active tDCS group. This effect was stronger after the second tDCS session. They proved that tDCS can decrease the total amount of morphine used. As a secondary objective, VAS pain scores were evaluated at specific time points: during rest, in cough, and during movement. The results showed that for the tDCS group, there was a decrease in VAS score with cough. No complications were noted due to the transcranial stimulation procedure. Interestingly, no differences between groups were noted regarding anxiety, mood, depression, or patient-related outcomes, somehow in discordance with studies on chronic pain [29].

The latest [30] randomized trial published in February 2023 came from Egypt and showed a statistical decrease in pain, as well as in the depression score in the group that received stimulation, pre vs. post treatment. Focusing on range of motion, shoulder flexion and extension increased by 4.8% and 5.5%, respectively, in the active stimulation group. Patients were evaluated before the first stimulation and after the last stimulation session, with no further follow-up. This is the only trial that used bilateral cranial stimulation, irrespective of the side of the pain.

#### 3.2.4. Conclusion Regarding tDCS in Cancer Pain

Considering the diversity of pain mechanisms and pathologies, the limited number of patients, and different stimulation parameters, no conclusion can be drawn regarding the efficacy of tDCS in cancer pain. This method is of major interest and is currently under research in this category of patients in a randomized controlled trial, STIMPAL [56], as well as in a Painless PanEuropean Horizon project [60]. A trial of tDCS involving survivors of pediatric bone sarcoma with chronic pain is also underway [NCT05746429]. Future data on better-represented cohorts will provide further information on the role of tDCS in cancer patients.

### 3.3. Cranial Electrical Stimulation

#### 3.3.1. Brief Technique Overview

Cranial electrical stimulation is another method of neuromodulation. It uses a pulsed current stimulation technique that modifies alpha and beta wave frequencies, increasing the concentration of neurotransmitters, thus having potential neuroplastic and cognitive effects. It does not polarize brain tissue but stimulates it in a rhythmic manner, with potential enhancement of the efficacy of endogenous neurophysiologic activity [32]. This method is recognized as a medical device that is used for the treatment of depression, anxiety, insomnia, and pain. These devices can deliver electrical stimulation through electrodes attached to earlobes. The intensity was <1 mA at 100 Hz. The Cochrane meta-analysis [14] included five studies using CES with 270 patients and showed no statistical effect of CES on pain.

#### 3.3.2. Studies Using CES in Cancer Pain

Through the search, three studies of CES in cancer patients were identified: a prospective, three-group, randomized, double-blinded, longitudinal pilot feasibility study [31], a randomized sham-controlled trial [32], and a non-randomized feasibility study [33]. For further details, refer to Table 3. All three studies had an IMMPACT recommendation score of four. None of the studies discussed below were included in the Cochrane analysis, whereas the 2023 meta-analysis by Chien [21] included Lyon’s study published in 2015 [32].

All three studies used the same Alpha-Stim Stress Control System and stimulation parameters. The first study [31] regarding CES dates back to 2010 and investigated the effect of CES on reducing the symptoms of depression, anxiety, fatigue, pain, and sleep disturbances. Their study was a randomized pilot feasibility trial applied to patients with breast cancer receiving chemotherapy. In the interest of concrete results, they used three groups: the active stimulation group, a sham group, and a group that received neither active nor sham. The period of usage was six or eight weeks depending on the timing of the chemotherapy protocols. The protocol was complex, using questionnaires and blood samples to test for inflammatory biomarkers. Their study showed that the method is feasible, as 72% of the eligible patients were enrolled. No adverse events were recorded and none of the participants reported stopping the procedure. The study showed that the devices and the method were safe and acceptable to patients. The results on anxiety, pain, depression, and fatigue were not statistically significant. Authors considered this to be due to the missing data and the disadvantages of the interactive voice response method used for data collection. No conclusion can be drawn from this study regarding the efficacy of CES for cancer pain [31].

The same American team continued their research on CES and published a new article in 2015 [32]. This protocol is somewhat related to the first protocol. They used the same questionnaires but only two groups: sham and active stimulation. Congruent with the first study, they found a certain level of symptoms in breast cancer patients, with fatigue and depression increasing over time. Pain and sleep disturbances fluctuated and anxiety levels decreased over time. Their results were non-significant. It was concluded that CES had no effect on the symptoms of patients with breast cancer during chemotherapy. Their explanation focused on the floor effect, as patients’ symptoms were not severe enough to benefit from the intervention [32].

The most recent study on CES [33] was designed as a preliminary study to test the feasibility and efficacy of CES in patients with advanced cancer. The protocol included several questionnaires and saliva samples. The adherence rate was 92%, which revealed good feasibility. Their results showed a statistically significant difference in several symptoms, including pain after the four weeks use of CES. Overall, their results are promising, but the lack of a control group is a major limitation [33].

#### 3.3.3. Conclusion Regarding CES in Cancer Pain

Congruent with the Cochrane meta-analysis [14] that examined non-oncologic patients, the studies discussed could not demonstrate the efficacy of CES in ameliorating pain in patients with cancer. Therefore, further studies are warranted. Currently, no trials involving CES in cancer patients are reported as open on clinicaltrials.gov. The impact of CES in this category of patients remains unknown.

## 4. Conclusions

Pain is a permanent challenge for oncologists, and new treatment options are constantly being researched. Non-invasive brain stimulation is a relatively new method of neuromodulation that has proven beneficial in relieving chronic pain. With few exceptions, trials regarding pain in patients with cancer were excluded from systematic reviews and guidelines. The role of NIBS in the management of pain in cancer patients remains undefined. There is a paucity of data, with only a handful of studies for each technique, using a limited number of patients and different stimulation parameters. This diversity makes it difficult for medical specialists to assess the potential benefits of NIBS and to integrate them into the therapeutic management of cancer pain. There is an urgent need for more data regarding non-invasive brain neuromodulation techniques in patients with cancer.

## Figures and Tables

**Table 1:** Studies of rTMS in the management of cancer-related pain.

**First author**		Khedr [24]		Khedr [25]		Goto [26]	Tang [27]	
**Country of origin**		Egipt		Egipt		Japan	China	
**Year of publication**		2015		2015		2020	2022	
**Type of study**		Randomized clinical trial.		Preliminary randomized trial.		Pilot randomized controlled, single-blind, four-way crossover study.	Randomized double-blind, parallel-group, sham-controlled, clinical trial.	
**IMMPACT Recommendations Score**		4/6		4/6		3/6	4/6	
**Type of pain**		Neuropathic pain with a score ≥ 4 on the Douleur Neuropathique 4 questionnaire.		Visceral pain.		Neuropathic pain with a minimum grade 2 severity based on the National Cancer Institute Common Terminology Criteria for Adverse Events (NCI-CTCAE, version 4.0) scale.	Lung cancer pain.	
**Inclusion criteria**		Age: 18–65 years; Malignant neuropathic pain resistant to medical treatments for at least 2 months.		Age: 18–65 years; Malignant visceral pain resistant to medical treatment for at least 2 months or associated with significant adverse effect from medication.		Age: >20 years; Any stage of cancer; Patients with a confirmed treatment plan consisting of taxane-based or oxaliplatin based chemotherapy; Neuropathic pain and/or peripheral sensory neuropathy; A performance status of Karnofsky 80%; 30 mm score in VAS of pain or dysesthesia intensity.	Age: 18–70 years; Confirmed diagnosis of advanced NSCLC by pathology or cytology; Pain symptoms and confirmed as cancer pain by oncologist; Experienced worst pain score ≥ 4 on NRS at the site of pain; Clear awareness and could cooperate to evaluate pain severity; Estimated that the survival time is more than 3 months; Completion of signed informed consents and voluntary participation in this study.	
**Exclusion criteria**		Intracranial metallic devices, pacemakers, or other devices; Recent myocardial ischaemia, unstable angina; History of epilepsy.		Intracranial metallic devices, pacemakers, or other devices; Extensive myocardial ischaemia, unstable angina; Epilepsy.		Implanted devices; History of seizures; Metal implants in the head; Higher brain dysfunction; Severe depression (>41 scores of the Beck Depression Inventory).	Brain tumour patients; History of seizure; Implanted pacemaker, stent, and other metal substances; Acute pain anywhere in the body due to other diseases; Serious psychiatric diagnoses (e.g., psychosis).	
**Period of study**		January 2010–May 2013		January 2010–January 2012		April 2015–October 2016	January 2020–March2021	
**Study patients**		Intervention group	Control group	Intervention group	Control group	Pilot study, no control group	Intervention group	Control group
**Initial number of patients**		17	17	17	17	11	21	21
**Final number of patients**		15	15	15	15	11	20	19
**Losses (no/%)**		2 (11.7%)	2 (11.7%)	2 (11.7%)	2 (11.7%)	0	1 (4.7%)	2 (9.5%)
**Age (years) mean ± SD**		47 ± 9.2	48 ± 9.7	51 ± 9.7	57.8 ± 3.9	64.8 ± 7.8	58.5 ± 8.9	59.6 ± 7.7
**Gender**								
**Male**		1	2	10	8	0	12	10
**Female**		16	15	7	9	11	8	9
**Duration of disease (months)**		15.4 ± 15.9	16.8 ± 16.3	15 ± 19.6	12.3 ± 14.9	Unknown	Unknown	
**Oncologic pathology/Cancer** **(no patients)**		Post mastectomy 14 Soft tissue sarcoma 1 Giant cell glioma 1 Femoral mass 1	Post mastectomy 15 Soft tissue sarcoma 2	Pancreas 4 Hepatic 7 Gall bladder 2 Stomach 1 NH lymphoma 1 Peritoneal mesothelioma 1	Pancreas 5 Hepatic 6 Gall Bladder 2 Stomach 2 Oesophagus 1 NH lymphoma 1	Breast 9 Gynecologic 2	Lung cancer 42	
**Treatment analgesic**		Tramadol 100 mg bid Pregabalin 75 mg bid Gabapentin 400 mg bid Amitriptyline 25 mg bid	Tramadol 100 mg bid Pregabalin 75 mg bid Gabapentin 400 mg bid Amitriptyline 25 mg bid	Tramadol 100 mg bid Scopolamina 20 mg tid Amitriptilina 25 mg bid	Tramadol 100 mg bid Scopolamina 20 mg tid Amitriptilina 25 mg bid	Unknown	Morphine sulfate controlled-release tablets or oxycodone hydrochloride sustained-release tablets	
**STIMULATION DETAILS**	**Target/** **Coil type/** **Orientation/** **Stimulation Frequency/** **Intensity/** **Time/**	Contralateral to pain M1 hand; F8 coil/AP; 20 Hz (10 s, 10 trains, with intertrain interval 30 s); 80% RMT; 10 min.		Contralateral side of pain M1 hand or dominant hemishpere for epigastric pain; F8 coil/AP; 20 Hz (10 s, 10 trains, with intertrain of 30 s); 80% RMT; 10 min.		5 Hz, 10 s, 10 trains, with intertrain of 50 s, F8 coil; Stimulation types: 1. PA 90%: PA coil direction with 90% RMT target M1 stimulation, 2. PA 100%: PA coil direction with 100% RMT target M1 stimulation, 3. Ipsilateral PA 90%: PA coil direction with 90% RMT stimulation ipsilateral to the target M1 (if the target M1 was “the right-hand area”, then ipsilateral target M1 was “the right leg area”), 4. LM 90%: LM coil direction with 90% RMT target M1 stimulation.	DLPFC left side; F8 coil; 10 Hz (15 pulse trains (1.5 s), with intertrain of 3 s); 80% RMT.	
	**No of pulses/sessions** **No of sessions**	2000 pulses; 10 sessions; daily; 2 weeks.		2000 pulses; 10 session; daily; 2 weeks.		500 pulses; 4 sessions in 2 months.	1500 pulses; 15 sessions; daily; 3 weeks.	
**Endpoints**		Primary VAS after 10th session and 1 month later; Secondary VDS, LANSS, and HAM-D after 10th session and 1 month later.		Primary VAS after 10th session and 1 month later; Secondary VDS and HAM-D after 10th session and 1 month later; Serum Dynorphin.		P-VAS; SF-MPQ2; D-VAS.	NRS; OME, Quality of life; HAM-A; HAM-D.	
**Adverse events**		No		No		No	two patients with transient scalp numbness or facial muscle twitching	
**Results**		Reduction in pain score at the end of rTMS protocol (49% on VRS and 36.7% on VAS). Effect maintained for 2 weeks with reduction (45.6% on VRS and 35.5% on VAS) but not at 1 month. Reduction in LANNS score at the end of rTMS protocol—21.9% and at 2 weeks—20.9%. Reduction in HAM-D at the end of rTMS protocol—24.4% and at 2 weeks—23.6%. Percentage of pain relief in the VAS scale; the number of responders (30% or more pain relief) were 13 (86.6%), 12 (80%), and 4 (26.6%) at the three time points of assessment in the real group.		Pain relief in malignant visceral pain for at least 15 days, with reduction in pain score at the end of rTMS protocol (35.6% on VRS and 30% on VAS) and at 2 weeks after the last session (39.7% on VRS and 26% on VAS). The effect is not maintained at one month. No significant differences in HAM-D. Serum human Dyn level shows no significant difference between patients with visceral pain treated with real rTMS and sham rTMS.		Amelioration in pain or disesthesia mainly in the targeted extremity. At the target extremity: – P-VAS scores significantly decreased after LM 90% (*p* = 0.03). – D-VAS scores significantly decreased after PA 90% (*p* = 0.03) and LM 90% (*p* = 0.04). – SF-MPQ2 score decreased significantly after PA 90% (*p* = 0.01). At the three non target extremities: – only D-VAS scores significantly decreased after PA 90% (*p* = 0.047).	Decrease in NRS in the treatment group starting from the 3rd day to the 3rd week (decrease of 41.09% in the rTMS group and 23.23% in the sham group). On week 3, the OME in the rTMS group was similar to that of baseline (*p* = 0.02), while the sham group was significantly higher than that of baseline (*p* = 0.02). The physiology and psychology domains of WHOQOL-BREF scores showed significant improvements with rTMS group versus sham group (*p* = 0.02). HAM-A and HAMD scores in the rTMS group showed significant improvements after 3 weeks of treatment when compared with baseline (*p* = 0.005 and *p* = 0.011).	

**Abbreviations:** rTMS, repetitive transcranial magnetic stimulation; NRS, numeric rating scale; VAS, visual analogue scale; VDS, verbal descriptor scale; SD, standard deviation; NSCLC, Non-Small Cell Lung Cancer; NHL, Non-Hodgkin Lymphoma; AP, antero-posterior; PA, postero-anterior; LM, lateral-medial; M1, motor cortex cortical area; RMT, resting motor threshold; DLPFC, dorsolateral prefrontal cortex; HAM-A, Hamilton anxiety rating scale; HAM-D, Hamilton depression rating scale; P-VAS, pain visual analogue scale; LANSS, Leeds assessment of neuropathic symptoms and signs; OME, oral morphine equivalent; SF-MPQ2, Short-form McGill Pain Questionnaire 2; D-VAS, Dysesthesia visual analogue scale; WHOQOL-BREF, World Health Organization Quality of Life-BREF.

**Table 2:** Randomized studies of tDCS in the management of cancer-related pain.

**First author**		Ibrahim [28]		Stamenkovic [29]		Hanna [30]	
**Country of origin**		Egipt		Serbia		Egipt	
**Year of publication**		2018		2020		2022	
**Type of study**		Prospective, randomized, double-blind sham-controlled.		Prospective, randomized, double-blind sham-controlled.		Prospective, randomized, sham-controlled.	
**IMMPACT Recommendations Score**		4/6		4/6		3/6	
**Type of pain**		HCC; Chronic abdominal/visceral pain.		Lung cancer; Acute pain post thoracotomy.		Post mastectomy pain syndrome scored ≥ 4 in DN4.	
**Inclusion criteria**		Age: >18 years; Chronic abdominal pain due to primary liver cancer or on top of cirrhosis that was resistant to medical treatment for at least 2 months or associated with significant adverse effects from medication.		Age: 18–80 years; Willingness to participate; Ability to understand the protocol and provide written informed consent; Scheduled thoracotomy for confirmed primary malignant lung disease and planned tracheal extubation in the operating room immediately after surgery.		Age: 25–69 years; Females with unilateral/single mastectomy, total mastectomy (with sentinel lymph node dissection), modified radical mastectomy (with axillary lymph node dissection), nipple-sparing mastectomy (with either sentinel or axillary lymph node dissection), and skin-sparing mastectomy; No or mild lymphedema; Female patients with PMPS; Presence of neuropathic pain post mastectomy surgery by DN4 questionnaire; Patients with neuropathic pain that lasted for 6 months or more.	
**Exclusion criteria**		History of chronic pain syndrome; Intracranial metallic devices or with cardiac pacemakers; Extensive myocardial ischemia; History of epilepsy.		Pregnancy; Treatment for neurological or psychiatric diseases; Any chronic pain condition; History of alcohol or drug abuse; Chemotherapy; History of previous thoracic or cardiac surgery; Allergy to medications used in the study; Presence of pacemaker, automatic implantable cardioverter/defibrillator, or any other implanted device in the head, spinal cord, or peripheral nerves; Confirmed brain lesion, including tumour or metastasis.		Epilepsy or a history of epilepsy or epileptic drugs; Intake; Medical diagnoses of psychological or neurological disorders; History of migraines; Scalp or skin condition (e.g., psoriasis or eczema); Metalic implants, including intracranial electrodes, surgical clips, shrapnel, or a pacemaker, or any metallic accessories or cloth; Head injury resulting in a loss of consciousness that has required further investigation (e.g., a brain scan); Seizure; Chance of pregnancy and patients on contraceptive pills; Moderate or severe lymphedema.	
**Period of study**		April 2015 to February 2016		15 June 2016 to 27 March 2018.		Unknown	
**Study patients**		Intervention group	Control group	Intervention group	Control group	Intervention grpup	Control group
**Initial number of patients**		24	24	30	31	20	18
**Final number of patients**		20	20	27	28	15	15
**Losses (no/%)**		4 (16%)	4 (16%)	3 (10%)	3 (9.6%)	5 (20%)	3 (6%)
**Age (years) mean ± SD**		58.9 ± 5.6	56.85 ± 9.16	61.44 ± 7.98	61.89 ± 5.79	40.5 ± 2.8	40.2 ± 3.1
**Gender**							
**Male**		14	13	16	23	0	0
**Female**		6	7	11	5	15	15
**Oncologic pathology**		HCC		Lung cancer		Breast cancer	
**Treatment analgesic**		Tramadol hydrochloride 50 mg twice daily.		Unknown		Unknown	
**Endpoints**		VAS; VDS; HAM-D.		Morphine dose; VAS—pain rest, movement, and couch; VAS—anxiety; Beck depression inventory; PRO.		VAS; Beck depression inventory; ROM.	
**Stimulation details**	**Anode location**	Primary motor cortex of the contralateral most painful abdominal area.		Left primary motor cortex.		Bilateral M1.	
	**Cathode location**	Contralateral supraorbital region.		Contralateral supraorbital region.		Supraorbital region.	
	**Stimulation details**	Duration: 10 sessions/2 weeks;		Duration: 5 daily sessions;		Duration: 5 daily sessions;	
		Intensity: 2 mA;		Intensity: 1.2 mA;		Intensity: 2 mA;	
		Duration of a session: 30 min;		Duration of a session: 20 min;		Duration of a session: 20 min × 2 (20 min each side);	
		Device: neuroConn Germany.		Device: neuroelectrics.		Device: unknown.	
**Side effects**		Slight burning sensation in three patients; Skin redness under the active electrode in two patients.		None declared.		None declared.	
**Outcome**		For the active stimulation group—reduction in VDS (*p* = 0.001) and VAS (*p* = 0.001) and HAM-D (*p* = 0.012); The effect started from the 5th session and continued to 1 month after stimulation, while in the sham group, the effect persisted for 5 days only.		Cumulative morphine dose in the first 120 h after surgery was significantly lower in the tDCS compared to sham group *p* = 0.043; On postoperative day 5, VAS pain score with cough was significantly lower in the tDCS group (*p* = 0.018) and pain interference with cough was 80% lower (*p* = 0.013).		In the stimulation group, there was a significant difference between pre- and post treatment mean values of: – Pain (*p* = 0.001). Pain decreased by 32% post treatment; – Depression index (*p* = 0.003): depression decreased by 3.7% post treatment; – Shoulder extension (*p* = 0.002): shoulder extension increased by 5.5% post treatment; – Shoulder flexion (*p* = 0.001): shoulder flexion increased by 4.8% post treatment.	

**Abbreviations**: tDCS, transcranial direct current stimulation; HCC, hepatocellular carcinoma; VAS, visual analogue scale; VDS, verbal descriptor scale; PRO, patient-reported outcomes; HAM-D, Hamilton Depression Scale; Beck, Beck Depression Inventory; ROM, range of motion; PMPS, post mastectomy pain syndrome; DN4, Douleur Neuropathique 4 questionnaire; SD, standard deviation; M1, primary motor cortex area.

**Table 3:** Studies of CES in the management of cancer-related pain.

**First author**	Lyon [31]			Lyon [32]		Yennurajalingam [33]
**Country of origin**	USA			USA		USA
**Year of publication**	2010			2015		2018
**Type of study**	Pilot feasibility study.			Randomized, sham-controlled trial.		Preliminary study, one group, open label.
**IMMPACT Recommendation** **Score**	4/6			4/6		4/6
**Inclusion criteria**	Stage I–IIIA breast cancer; Female; Age 18 years or older; Able to read and speak English; Scheduled to receive at least four cycles of an anthracycline-containing chemotherapy regimen.			Stage I–IIIA breast cancer; Performance score ECOG < 2; Scheduled to receive at least four cycles of adjuvant or neoadjuvant chemotherapy.		Advanced cancer; One or more of the four symptoms (depression, anxiety, sleep disturbance, and pain) with average intensity of ≥3/10 on the Edmonton Symptom Assessment Scale.
**Exclusion criteria**	Major psychiatric conditions; Treatment with antidepressants or anxiolytics; Implanted devices (cardiac pacemakers).			Previous chemotherapy; Dementia; Active psychosis; History of seizure disorder; Any implanted electrical device, Began or changed a medication regimen for depression or other psychiatric condition within 30 days prior to study enrolment.		Systemic anti-inflammatory medications; Mental illness (schizophrenia, bipolar disorder); Delirium (Memorial Delirium Assessment Scale (MDAS) score ≥ 7); Participating in other structured behavioural intervention(s); Pregnancy; Presence of an implantable device (pacemaker); Cancer of the head and/or neck or brain tumour or brain metastasis; A history of seizure.
**Stimulation details**	Alpha-Stim Stress Control System			Alpha-Stim Stress Control System		Alpha-Stim M
	Intensity: 100 μA;			Intensity: 100 μA;		Intensity: 100 μA;
	Frequency: 0.5 Hz;			Frequency: 0.5 Hz;		Frequency: 0.5 Hz;
	Duration: 60 min;			Duration: 60 min;		Duration: 60 min;
**Period of stimulation**	Daily for 6–8 weeks depending on chemotherapy schedule.			Daily for the chemotherapy period and 2 weeks after; (6–32 weeks).		Daily for 4 weeks.
**Outcomes**	Feasability; HADS; BPI; BFI; GSDS.			HADS; BPI; BFI; GSDS.		ESAS; HADS; PSQI; BPI; MDAS; NCCN Distress Thermometer Safety.
**Groups**	Active	Sham	Standard	Active	Sham	Not sham-controlled
**Number of patients initially**	36			84	83	36
**Number of patients analyzed**	13	10	12	77	75	33
**Losses number (%)**	0%	0%	0%	7 (8.33%)	8 (10.6%)	3 (8.33%)
**Age (years) ± SD**	47.54 ± 9.1	46.6 ± 5.64	50.5 ± 18.28	51.04 ± 1.21	51.91 ± 0.97	59
**Results**	Feasability of CES; No significant data regarding symptom relief.			No benefit of CES on depression, anxiety, pain, fatigue, and sleep disturbances.		Feasible and safe; Improvement in pain BPI pain (*p* = 0.013); Improvement in PSQI daytime dysfunction (*p* = 0.002); Improvment in ESAS anxiety (*p* = 0.001); ESAS depression (*p* = 0.025); Improvement in HADS depression (*p* = 0.024).

**Abbreviations:** CES, cranial electric stimulation; HADS, Hospital Anxiety and Depression Scale; ECOG, Eastern Cooperative Oncology Group Performance Status; BPI, Brief Pain Inventory; BFI, Brief Fatigue Inventory; GSDS, General Sleep Disturbance Scale; ESAS, Edmonton Symptom Assessment Scale; PSQI, Pittsburgh Sleep Quality Index; MDAS, Memorial Delirium Assessment Scale.

## Supplementary Materials

The following supporting information can be downloaded at: https://www.mdpi.com/article/10.3390/medicina59111957/s1, Table S1: Major databases search strategies.
