# Supplementary material for: Cancer Pain and Non-Invasive Brain Stimulation—A Narrative Review
Source: Medicina (Kaunas). 2023 Nov 6;59(11):1957. doi: 10.3390/medicina59111957 (PMC10673188; doi:10.3390/medicina59111957)
Supplement: Supplementary file 1 [file medicina-59-01957-s001.zip › medicina-2645733-supplementary.pdf]

Supplementary Table S1. Major databases search strategies.

Web of science	(TS=(cranial electrical stimulation) OR TS=(noninvasive cortical stimulation)OR TS=(noninvasive brain stimulation) OR TS=(transcranial magnetic stimulation) OR TS=TMS OR TS=(Transcranial direct current stimulation) OR TS=(Transcranial DC stimulation) OR TS=(Transcranial electric stimulation) OR TS=(Transcranial electrical stimulation) OR TS=(Transcranial cathodal stimulation) OR TS=(Transcranial anodal stimulation) OR TS=tDCS OR TS=(tDCS anodal) OR TS=(tDCS cathodal) OR TS=(Transcranial electrical current stimulation) OR TS=(Transcranial electric current stimulation)) AND (TS=pain) AND (TS= cancer OR TS= malignancy OR TS=malignant)
Pubmed	#1 transcranial magnetic stimulation[MeSH Terms] OR transcranial magnetic stimulation[Title/Abstract] OR magnetic stimulation transcranial[Title/Abstract] OR TMS[Title/Abstract] OR transcranial direct current stimulation[MeSH Terms] OR transcranial direct current stimulation[Title/Abstract] OR tDCS [Title/Abstract] OR electrical stimulation transcranial[Title/Abstract] OR electrotherapy stimulation[Title/Abstract]<br>#2 ("Pain"[Mesh] OR "Pain Management"[Mesh] OR "Chronic Pain"[Mesh] OR "Visceral Pain"[Mesh]) OR (pain[Title/Abstract])<br>#3 (((("Neoplasms"[Mesh] OR "Cancer Pain"[Mesh])) OR (cancer[Title/Abstract])) OR (malignant[Title/Abstract])) OR (malignancy[Title/Abstract])<br>#1 AND #2 AND #3
Cochrane	1.MeSH descriptor: [Transcranial Magnetic Stimulation] explode all trees OR "Transcranial Magnetic Stimulation" OR TMS OR MeSH descriptor: [Transcranial Direct Current Stimulation] explode all trees OR “Transcranial Direct Current Stimulation" OR tDCS OR MeSH descriptor:[Electric stimulation] explore all trees.<br>2.MeSH descriptor: [Pain] explore all trees OR pain<br>3.MeSH descripor: [Neoplasms] explore all trees OR cancer<br>#1 AND #3 AND #4
Scopus	(TITLE-ABS(transcranial direct current stimulation) OR TITLE-ABS(Transcranial magnetic stimulation) OR TITLE-ABS(cranial electrical stimulation)) AND TITLE-ABS(pain) AND TITLE-ABS(cancer).

Abbreviations: TS, Topic; MeSH,Medical Subject Headings; Abs, Abstract.
